# Predictors for infection severity for open tibial fractures: major trauma centre perspective

**DOI:** 10.1007/s00402-023-04956-1

**Published:** 2023-07-07

**Authors:** James Zhang, Victor Lu, Andrew Kailin Zhou, Anna Stevenson, Azeem Thahir, Matija Krkovic

**Affiliations:** 1 Department of Trauma and OrthopaedicsAddenbrooke’s Major Trauma Unit, Cambridge University Hospitals, Cambridge, UK; 2 School of Clinical Medicine, University of Cambridge, Cambridge, CB2 0SP UK

**Keywords:** Osteomyelitis, Tibial fractures, Open fractures, Superficial infection, Major trauma centre

## Abstract

**Introduction:**

Open diaphyseal tibial fractures are the most common long-bone fractures and require a rapid approach to prevent devastating complications. Current literature reports the outcomes of open tibial fractures. However, there is no robust, up-to-date research on the predictive indicators of infection severity in a large open tibial fracture patient cohort. This study investigated the predictive factors of superficial infections and osteomyelitis in open tibial fractures.

**Materials and methods:**

A retrospective analysis of the tibial fracture database was carried out from 2014 to 2020. Criteria for inclusion was any tibial fracture including tibial plateau, shaft, pilon or ankle, with an open wound at the fracture site. Exclusion criteria included patients with a follow-up period of less than 12 months and who are deceased. A total of 235 patients were included in our study, of which 154 (65.6%), 42 (17.9%), and 39 (16.6%) developed no infection, superficial infection, or osteomyelitis, respectively. Patient demographics, injury characteristics, fracture characteristics, infection status and management details were collected for all patients.

**Results:**

On multivariate modelling, patients with BMI > 30 (OR = 2.078, 95%CI [1.145–6.317], *p = *0.025), Gustilo-Anderson (GA) type III (OR = 6.120, 95%CI [1.995–18.767], *p = *0.001), longer time to soft tissue cover (*p = *0.006) were more likely to develop a superficial infection, and patients with wound contamination (OR = 3.152, 95%CI [1.079–9.207], *p = *0.036), GA-3 (OR = 3.387,95%CI [1.103–10.405], *p = *0.026), longer to soft tissue cover (*p = *0.007) were more likely to develop osteomyelitis.

Univariate analysis also determined that risk factors for superficial infection were: BMI > 35 (OR = 6.107, 95%CI [2.283–16.332], *p = *0.003) and wound contamination (OR = 2.249, 95%CI [1.015–5.135], *p = *0.047); whilst currently smoking (OR = 2.298, 95%CI [1.087–4.856], *p = *0.025), polytrauma (OR = 3.212, 95%CI [1.556–6.629], *p = *0.001), longer time to definitive fixation (*p = *0.023) were for osteomyelitis. However, none of these reached significance in multivariate analysis.

**Conclusion:**

Higher GA classification is a significant risk factor for developing superficial infection and osteomyelitis, with a stronger association with osteomyelitis, especially GA 3C fractures. Predictors for superficial infection included BMI and time to soft tissue closure. Time to definitive fixation, time to soft tissue closure, and wound contamination were associated with osteomyelitis.

## Introduction

Long bone fractures occur at a frequency of 11.5 per 100,000 person-years, with open tibial diaphysis fractures being the most common [1, 2]. 24% of all tibial fractures are open [1, 3]. Tibial fractures have a higher risk of being open than other long bones due to the limited soft tissue surrounding the tibia [1, 2]. These fractures typically occur bimodally: young males sustain high-impact injuries, and the elderly sustain low-energy fragility fractures [4]. The Gustilo-Anderson (GA) classification is widely used to classify open tibial fractures, with 3C fractures being the most severe and requiring vascular involvement [5, 6]. Open tibial fractures have surgical challenges due to poor soft tissue coverage, bone defects and high contamination risk [6]. This high contamination risk can lead to the development of osteomyelitis, with reports of open tibial fracture infection rates being as high as 29% [7–10]. Furthermore, the development of osteomyelitis will impose additional challenges and causes devastating consequences, including non-union, osteonecrosis, and amputation. Various other complications have also been reported in the literature [11–14].

Open long bone fractures are an orthopaedic emergency as they have catastrophic consequences if not managed rapidly and effectively [15–17]. Acute management is according to the British Orthopaedic Association Standards for Trauma and Orthopaedics (BOAST) 4 criteria, a collaboration between the British Association of Plastic, Reconstructive and Aesthetic Surgeons (BAPRAS) and BOAST [18]. General management principles include a timely multi-disciplinary approach, rapid administration of intravenous prophylactic antibiotics, realignment and splinting of the limb, meticulous wound debridement and soft tissue cover within 72 h after injury [18].

Current reports discuss the epidemiology of open tibial fractures, and they have investigated the short and long-term outcomes of post-traumatic open tibial fractures [2, 12]. However, there has yet to be an up-to-date robust report on the prognostic indicators of infection severity from a large cohort of open tibial fractures.

In this study, we sought to answer:

1. What is the current incidence of the development of superficial infection to deep infections in open tibial fractures?
2. Are patient and fracture characteristics predictive of superficial and deep infections in open tibial fractures?
3. Are environmental and temporal factors predictive of superficial and deep infections in open tibial fractures?

## Methods

### Patient cohort

A retrospective analysis of the Epic systemsTM database at a single trauma centre was carried out from September 2014 to September 2020. The inclusion criteria were any patient with a tibia fracture, including the tibial plateau, shaft, pilon or ankle, with an open wound at the fracture site. Exclusion criteria were any patients that had a follow-up of fewer than 12 months or were deceased. This yielded 268 open tibial fractures from a major trauma centre database. Three patients were transferred to other hospitals for treatment after admission to the emergency department. Eighteen patients had incomplete notes or were lost to follow-up, and twelve patients passed away within a month of injury due to complications from the original injury trauma. A total of 235 patients were included in our study, of which 159 (65.6%), 45 (17.9%), and 39 (16.6%) had no infection, superficial infection, or osteomyelitis, respectively.

### Definitions and coding

Patients were grouped according to infection outcomes. *Superficial infection* was defined as cellulitis over the fracture site, soft tissue abscesses, or positive microbiology samples from the tissue around the injury site, in addition to clinical signs of infection. *Osteomyelitis* was defined as clinical or radiological signs of osteomyelitis, in addition to positive bone debridement microbiology samples on debridement, infective non-union, infective bone loss or infective malunion or infected metalwork. Patients that did not fit the criteria above were classified as having *“no infection”.*

Mode of injury was classified into broad categories: falls, road traffic accidents, and crushes (including struck-by-a vehicle and pedestrian accidents). Definitive fixation was defined as the final operation to fix the fracture, including open reduction internal fixation (ORIF), external fixation (fine wire or with pins, FWF), non-operative joint fusion and amputation. The time to definitive soft tissue cover was defined as the time from admission to when the most thorough final soft tissue cover was achieved. Soft tissue cover included wound dressing (saline-soaked gauze), vacuum-assisted closure, skin graft and skin flap.

### Data collection

Patient, fracture, and wound characteristics were collected for all included patients, and management details and infection outcomes were also included. Patient characteristics included age, gender, smoking status, diabetic status and body mass index (BMI). Fracture characteristics included injury mode, time of injury, presence of polytrauma, fibula fracture status, and GA classification. Wound characteristics included wound contamination. Management details included time to the first procedure, time to definitive soft tissue cover, time to definitive fixation and type of definitive fixation. Infection status was collected according to our definition above, and then patients were grouped into no infection, superficial infection and osteomyelitis.

### Data analysis

Statistical analysis was performed by univariate analysis and multicollinearity diagnostics, followed by multivariate model generation, with PSS v28.0 being used. Univariate analysis was conducted with odds ratios for binary categorical independent variables, and bivariate regression for continuous independent variables for both non-infection versus superficial infection, as well as non-infection versus osteomyelitis.

A cut-off of *p < *0.2 was used to determine which independent variables in the univariable calculations to include in the multivariable modelling, for both “no infection vs superficial infection” and “no infection vs osteomyelitis” comparisons. Each variable for inclusion in the multivariate model had its variance inflation factor (VIF) checked, to ensure there was no strong multicollinearity between the independent variables that could impact the validity of the multivariate model. A cut-off of VIF > 5 was used to exclude variables.

Multivariable analysis was performed to elucidate which variables were significant for “non infection vs superficial infection” and “non infection vs osteomyelitis”, with *p < *0.05 considered statistically significant.

For categorical variables with more than two possible groups, a combination strategy was performed to achieve clinically relevant groupings for odds ratio computations, e.g. for smoking (current smoker comparison: ‘never smoked + ex-smoker vs smoker, have ever smoked comparison: never smoked vs ‘ex-smoker + current smoker’).

For continuous variables, the entire range was divided into groups when appropriate, e.g. for age, a cut-off of 60 years was used, and for BMI, the clinical designations of classes (normal weight, overweight, obese) were used. For time to procedures, no obvious cut-offs or groupings were available, so they were only analysed directly in the multivariate model.

## Results

### Patients and injury characteristics

The demographic data breakdown of all three infection groups is shown in Table 1.

**Table 1:** Demographic features and injury characteristics across the groups

Factor	Subcategory	Total	No infection	Superficial infection	Deep infection
Number of patients		235	154	42	39
Age (mean)		55.2 (13–103)	55.6 (13–93)	58.7 (13–93)	49.9 (20–89)
Gender					
	Male	128	84	24	19
	Female	108	70	18	20
Mode of injury					
	Fall	141	104	23	14
	Road traffic accident	78	40	16	22
	Crush/hit	16	10	3	3
Polytrauma					
	No	165	118	28	19
	Yes	70	36	14	20
Fibula fractured					
	No	77	53	14	10
	Yes	158	101	28	29
Gustilo-Anderson Classification type					
	I	42	36	2	4
	II	46	42	3	1
	IIIa	57	36	12	9
	IIIb	66	35	17	14
	IIIc	24	5	8	11
Location of fracture					
	Plateau	7	2	1	4
	Shaft	14	5	2	7
	Pilon	111	70	22	19
	Ankle	103	77	17	9
Wound contamination					
	No	192	135	31	26
	Yes	43	19	11	13
Hours to first procedure		15.7 (0–403)	13.8 (0–79)	12.5 (1–49)	27.3 (1–403)
Hours to definitive soft tissue coverage		91.3 (1–730)	51.8 (1–528)	155.2 (1–730)	178.6 (1–984)
Type of definitive fixation					
	ORIF	123	101	14	8
	FWF	87	37	24	26
	Amputation	17	9	4	4
	Non-operative	4	4	0	0
	Ankle fusion	4	3	0	1
Hours to definitive fixation		631.4 (1–18,960)	354.6 (1–10,800)	913.1(1–18,960)	1435.1(1–17,520)
Smoking status					
	Non smoker	142	101	22	19
	Ex-smoker	38	22	11	5
	Current smoker	55	31	9	15
Body Mass Index		27.1 (16.4 – 48.7)	26.8 (17.6–48.5)	29.7(16.4–48.7)	25.2(16.6–35.1)
Diabetes					
	No	212	140	37	35
	Yes	23	14	5	4

*ORIF* open reduction internal fixation, *FWF* fine wire fixator, *BMI* body mass index

### Predictors of osteomyelitis, superficial infection, and no infection

Patients who had GA type III fractures, wound contamination, longer time to soft tissue cover, and BMI over 30, especially if over 35, were significantly more likely to develop a superficial infection (Table 2). Patients with GA type III, wound contamination, polytrauma, longer time to soft tissue cover, longer time to definitive fixation, or currently smoking are more likely to develop osteomyelitis (Table 3).

**Table 2:** Univariable analysis of demographic, injury and treatment factors to determine risk factors for superficial infection

Factor	Odds ratio	*p* value	95% Confidence interval
Univariate analysis of risk factors for superficial infection			
Age over 60	1.216	0.34	0.627–2.36
Female gender	0.766	0.272	0.391–1.501
Have ever smoked	1.821	0.056	0.934–3.55
Current smoker	1.189	0.4	0.546–2.591
Overweight (BMI > 25)	1.354	0.244	0.682–2.691
Obese (BMI > 30)	**2.941**	**0.002**	**1.461–5.92**
Morbidly obese (BMI > 35)	**6.107**	** < 0.001**	**2.283–16.332**
Polytrauma	1.683	0.106	0.828–3.422
Fibula fractured	0.854	0.403	0.42–1.738
Wound contamination	**2.249**	**0.047**	**0.985–5.135**
Gustilo-Anderson Type III	**8.308**	** < 0.001**	**3.117–22.142**
Hours to first procedure		0.572	
Hours to soft tissue cover		** < 0.001**	
Hours to definitive fixation		0.127	

Bold = significant at *p < *0.05

*BMI* body mass index

**Table 3:** Univariable analysis of demographic, injury and treatment factors to determine risk factors for deep infection

Factor	Odds ratio	*p* value	95% Confidence interval
Univariate analysis of risk factors for osteomyelitis			
Age over 60	0.565	0.092	0.267–1.195
Male gender	1.209	0.362	0.66–2.437
Have ever smoked	1.822	0.066	0.905–3.714
Current smoker	**2.298**	**0.025**	**1.087–4.856**
Overweight (BMI > 25)	0.71	0.218	0.352–1.433
Obese (BMI > 30)	0.804	0.41	0.327–1.981
Morbidly Obese (BMI > 35)	0.497	0.441	0.06–4.093
Polytrauma	**3.212**	**0.001**	**1.556–6.629**
Fibula fractured	0.652	0.191	0.296–1.4362
Wound contamination	3.614	0.003	1.595–8.188
Gustilo-Anderson type III	**7.062**	** < 0.001**	**2.627–18.984**
Hours to first procedure		0.153	
Hours to soft tissue cover		** < 0.001**	
Hours to definitive fixation		**0.023**	

Bold = significant at *p < *0.05

*BMI* body mass index

A threshold of *p < *0.2 was used to determine which variables from univariate analysis would be considered for inclusion in the multivariate model. After multicollinearity diagnostics for both models, none of the remaining variables was excluded, as the VIF did not reach 5 for any of them. The multivariate models for superficial infection and osteomyelitis are displayed in Tables 4 and 5, respectively.

**Table 4:** Multivariable analysis of demographic, injury and treatment factors to determine risk factors for superficial infection

Factor	Odds ratio	*p* value	95% Confidence interval
Multivariate analysis of risk factors for superficial infection			
Have ever smoked	1.209	0.731	0.410–3.565
Current smoker	0.951	0.940	0.258–3.501
Obese (BMI > 30)	2.078	0.197	0.684–6.317
Morbidly Obese (BMI > 35)	**4.990**	**0.032**	**1.145–21.737**
Polytrauma	1.234	0.662	0.482–3.160
Wound contamination	2.039	0.196	0.692–6.007
Gustilo-Anderson type III	**6.120**	**0.002**	**1.995–18.767**
Hours to soft tissue cover		**0.043**	
Hours to definitive fixation		0.390	

Bold significant at *p < *0.05

*BMI* body mass index

**Table 5:** Multivariable analysis of demographic, injury and treatment factors to determine risk factors for deep infection

Factor	Odds ratio	*p* value	95% Confidence interval
Multivariate analysis of risk factors for osteomyelitis			
Age over 60	0.553	0.330	0.168–1.819
Have ever smoked	1.404	0.641	0.338–5.841
Current smoker	0.971	0.982	0.199–4.732
Polytrauma	**3.604**	**0.030**	**1.135–11.442**
Fibula fractured	**0.261**	**0.032**	**0.077–0.890**
Wound contamination	2.135	0.199	0.672–6.782
Gustilo-Anderson Type III	**2.451**	**0.026**	**1.568–7.692**
Hours to first procedure		0.117	
Hours to soft tissue cover		**0.020**	
Hours to definitive fixation		0.331	

Bold = significant at *p < *0.05

On multivariate modelling, patients with BMI > 30, GA type III, and longer time to soft tissue cover were more likely to develop a superficial infection. Patients with wound contamination at injury, GA type III and longer time to soft tissue cover were more likely to develop osteomyelitis.

## Discussion

This study involved a large-scale analysis of risk factors for infection severity in open tibia fractures. Infections were stratified into superficial infection and osteomyelitis, and we found that BMI was associated with superficial infection, whilst time to definitive fixation, time to definitive soft tissue cover, and wound contamination was associated with osteomyelitis. GA classification was associated with both superficial infection and osteomyelitis, with more severe forms having a stronger association with osteomyelitis.

### Early debridement of open tibia fractures

The ideal treatment protocol for open tibia fractures is still controversial, however, all agree upon the need for irrigation and debridement [19–21]. Some studies, for example, Pollak et al. have reported no correlation between time of care, including debridement and rate of infections or infection severity [22, 23]. However, if they were admitted within 6 h post-injury, they had a lower infection rate [23]. This is supported by a historic precedent/ evidence of a ‘6-h’ window that debridement must be performed within. However, this is largely unsubstantiated [24–26], as only one study with 47 open tibia fractures concluded that surgical debridement within 5 h was associated with fewer infections [27]. However, they did not consider that their cohorts had different injury severity. Some studies have also suggested that earlier debridement leads to worse outcomes. However, these were biased toward treating the most severe cases earliest [28]. Contamination load and overall bacterial growth time are important determinants of infection severity. For example, caseating necrotic wound diameter grows steadily within hours of bacterial contamination, with virulent strains leading to a more rapid increase in bacterial count, especially in deep wounds with low oxygen partial pressures and in areas with poor blood supply like the tibia [29]. After adjusting for confounding factors such as fracture type and injury grade, our cohort showed that the time to definitive soft tissue cover and definitive fixation was associated with the risk of developing osteomyelitis, suggesting the importance of timely management of open tibia fractures. Given the fact that it is unethical to randomise patients with severe trauma based on time to treatment, prospective cohort-style studies may still be the most practical in investigating the relationship between time to treatment and outcomes.

### Correlation of Gustilo-Anderson classification to infection and poorer outcomes

Our study utilised the Gustilo-Anderson classification for open tibia fractures, despite one study reporting an average interobserver agreement of 60% [30]. The original classification system by Gustilo did not subdivide type III fractures. However, this was eventually amended after Gustilo et al. realised that the incidence of complications could not be accurately determined [31]. Gaebler et al. suggested that type III GA fractures led to a higher chance of deep infection. Nevertheless, only 43.3% of their patients had open fractures, and they did not subdivide the type III fractures, which limits the usefulness of their results in open tibia fracture research [32]. Schemitsch et al. reported many risk factors associated with poorer prognosis after intramedullary nailing of open tibia fractures, such as whether or not the nail was reamed. However, they failed to include GA classification in their analysis and simply excluded type IIIc open fractures [33]. Our study found a significant relationship between GA classification and infection severity, which agrees with many retrospective cohort studies in the literature. Harley et al. determined that a strong predictor for infection was GA type (type I had a 2% rate; type III had a 22% rate) [34]. However, studies like these are confounded by the significant number of comorbidities in open tibia fracture patients, such as diabetes, intra-operative factors such as blood loss and operative time, as well as external factors such as tobacco and alcohol use, all of which can influence functional and clinical outcomes [17, 35]. Furthermore, the type of definitive fixation performed on open tibial fracture patients, namely intramedullary nails (IMN), screws and plating, or external fixation with frames, has an impact on rates of infection as well as overall complication rates [36]. The patient data we received showed predominantly IMNs for definitive fixation, which could explain our lower infection rates when compared to the literature. IMNs fixation has been associated with superior post-operative outcomes and is the preferred mode of definitive internal fixation amongst trauma surgeons [37, 38].

### Correlation between body habitus and infection

BMI was found to be associated with superficial infections but not osteomyelitis. In a national prospective cohort study involving 159,720 patients, obesity was linked to a 1.1–4.4 fold increase in developing a superficial infection compared with normal weight [39]. The reasons for this are multifactorial. Obesity is associated with a chronic inflammatory state, and metabolic factors such as increased insulin resistance and hyperglycaemia that are characteristic of obese patients could render patients susceptible to infections [40]. Obese patients could have different injury patterns after blunt and severe trauma [41], coupled with prolonged recovery and immobility after trauma. They are more likely to have central lines inserted for longer periods of time due to difficulty with peripheral venous access [42]. Furthermore, mechanical factors can increase the superficial infection rate. These include increased local surgical site trauma due to increased tissue retraction and adipose tissue content; prolonged operative times; and decreased perioperative tissue oxygenation [43].

### Deep infections vs osteomyelitis

The literature is currently unclear on the definition of osteomyelitis and what is necessary for its diagnosis. Most papers have a variety of criteria and require one or more criteria to be met. These criteria can include signs on imaging [44–47]; positive cultures or histology from bone biopsies or debridement [46–48]; or sinuses or abscesses during surgery [44–46]. The emphasis on which of these findings is essential vs optional varies by paper. We, therefore, decided to use a definition which was based on the majority of papers we read which supported that microbiological results are preferable [46–48].

### Limitations

Being a retrospective observational study, we relied upon the accuracy of patient’s notes and attendance at follow-up clinics. The single-centre nature of the study also can restrict the scope of patients treated. However, being a major trauma centre with a dedicated bone infection team means that there will be good variation and diversity of patients and they are unlikely to be followed up in other settings. This, combined with the wide range of patient factors studied and the long-time frame investigated enhances the external validity of our results. With traumatic injury cases, investigating fixation methods in a prospective manner, negating the impact of the specific situation and surgeons’ experiences, can be difficult to justify ethically and implement. Nevertheless, future studies involving multiple centres could further elucidate underlying factors for infection.

In our study, we grouped various anatomical locations of tibial fractures, such as ankle, plateau, pilon and shaft, together into the same cohort. While there is inherent heterogeneity within this group, we feel that for open fractures specifically, this is justified given the underlying mechanisms of infection (e.g. wound contamination, vasculature and subsequent post-injury compromise, and delay in wound closure) are quite similar within that specific area of the lower limb.

Lastly, we classified fractures by GA type, rather than by AO classification. We felt this was appropriate given the degree of soft tissue damage is a stronger predictor of infective processes at the time of injury, rather than the degree of comminution or articular involvement. Furthermore, with AO classification more commonly used in research activities rather than clinical practice, we feel that the GA type is more suitable for clinicians when risk stratifying at the time of injury.

## Conclusion

Open tibial fractures are the most common open long bone fractures and are notorious for developing infections during rehabilitation. Our study demonstrated GA classification is a significant indicator for both superficial infection and deep infection, with a stronger association with osteomyelitis, especially GA IIIC fractures. Other predictors for superficial infections included BMI and type of definitive fixation. Furthermore, time to definitive fixation, type of definitive fixation, time to definitive soft tissue cover, and wound contamination was associated with osteomyelitis. As FWF is typically used for patients with more complex fractures, they have a significantly increased chance of osteomyelitis and superficial infection compared to those with ORIF for definitive fixation. Patients who undergo ORIF tend to have faster definitive soft tissue closure and reduced contact with the external environment from metalwork. However, determining the definitive fixation method is difficult to do validly and ethically, and thus we recommend surgeons employ their own expertise in the area combined with the risk stratification of other factors reported in our study. Furthermore, it is pertinent that the BOAST 4 guidelines are followed and practitioners provide rapid intravenous prophylactic antibiotics to patients with wound contamination, a history of smoking or increased BMI to decrease the risk of developing infection.
